# Does Nordic Walking restore the temporal organization of gait variability in Parkinson’s disease?

**DOI:** 10.1186/s12984-017-0226-1

**Published:** 2017-02-21

**Authors:** Thibault Warlop, Christine Detrembleur, Maïté Buxes Lopez, Gaëtan Stoquart, Thierry Lejeune, Anne Jeanjean

**Affiliations:** 1 Physical and Rehabilitation Medicine Department, Cliniques universitaires Saint-Luc, Avenue Hippocrate n°10, 1200 Brussels, Belgium; 2 Institut de Recherche Expérimentale et Clinique, Neuro Musculo Skeletal Lab (IREC/NMSK), Université catholique de Louvain, Brussels, Belgium; 3 Louvain Bionics, Université catholique de Louvain, Brussels, Belgium; 4 Institut Parnasse-Deux Alice, Brussels, Belgium; 5 Institute of Neurosciences (IoNS), Université catholique de Louvain, Brussels, Belgium; 6 Neurology Department, Cliniques universitaires Saint-Luc, Brussels, Belgium

**Keywords:** Parkinson's disease, Gait disorders, Hypokinesia, Accelerometers, Locomotion, Rehabilitation, Gait variability, Nonlinear dynamics, Fractals

## Abstract

**Background:**

Gait disorders of Parkinson’s disease (PD) are characterized by the breakdown of the temporal organization of stride duration variability that was tightly associated to dynamic instability in PD. Activating the upper body during walking, Nordic Walking (NW) may be used as an external cueing to improve spatiotemporal parameters of gait, such as stride length or gait variability, in PD. The aim of this study was to evaluate the beneficial effects of NW on temporal organization of gait variability and spatiotemporal gait variables in PD.

**Methods:**

Fourteen mild to moderate PD participants and ten age-matched healthy subjects performed 2 × 12 min overground walking sessions (with and without pole in a randomized order) at a comfortable speed. Gait speed, cadence, step length and temporal organization (i.e. long-range autocorrelations; LRA) of stride duration variability were studied on 512 consecutive gait cycles using a unidimensional accelerometer placed on the malleola of the most affected side in PD patients and of the dominant side in healthy controls. The presence of LRA was determined using the Rescaled Range Analysis (Hurst exponent) and the Power Spectral Density (α exponent). To assess NW and disease influences on gait, paired *t*-tests, Z-score and a two-way (pathological condition x walking condition) ANOVA repeated measure were used.

**Results:**

Leading to significant improvement of LRA, NW enhances step length and reduces gait cadence without any change in gait speed in PD. Interestingly, LRA and step length collected from the NW session are similar to that of the healthy population.

**Conclusion:**

This cross-sectional controlled study demonstrates that NW may constitute a powerful way to struggle against the randomness of PD gait and the typical gait hypokinesia. Involving a voluntary intersegmental coordination, such improvement could also be due to the upper body rhythmic movements acting as rhythmical external cue to bypass their defective basal ganglia circuitries.

**Ethics committee’s reference number:**

B403201318916

**Trial registration:**

NCT02419768

## Background

Scaling and timing internal control required for automatic and rhythmical movements are impaired and archetypally linked to the basal ganglia dysfunction in Parkinson’s disease (PD). As a result of such impairment, a reduced gait speed, shorter stride length, reduced arm swing and a random walking pattern (i.e. increased gait variability) are typical features of PD gait [1–5]. From the specific temporal point of view, the inability to produce a steady gait rhythm, which result in more random stride-to-stride variability, is one of the primary temporal gait disorders and can indicate a sensitive marker of a higher fall risk in PD [5, 6].

Interestingly, the randomness of PD gait was recently emphasized by subtle deterioration of the temporal organization of gait variability, using the long-range autocorrelations (LRA) assessment [6]. In addition, close correlations with disease severity and balance status were demonstrated in PD [6]. In a more theoretical framework, LRA have indeed been suggested as an indicator of adaptive abilities characterizing healthy systems [5, 7–9]. As a corollary, deviations from an optimal level of variability in either the direction of randomness or the over-regularity are thought to reflect the loss of the adaptive capabilities of the system [8].

Consequently, interventions that improve LRA might be beneficial in PD gait, which is characterized by less efficient adaptive resources. Among numerous rehabilitative approaches, Nordic Walking (NW) holds a special place in PD as an emerging and promising strategy to stimulate an active lifestyle [10]. Using specially designed poles, NW involves an intentional coordination between upper and lower limbs and could constitute an interesting option to enhance the stride length and the gait speed [11–13]. Furthermore, the use of Nordic poles may act as an external cue, triggering intact circuits and bypassing the defective basal ganglia—SMA loop. This permits compensating for the impaired scaling and timing control in PD [12].

However, it remains unknown whether Nordic Walking, without any long-term training, can influence real-time spatial and temporal gait variables in Parkinson’s disease, and especially long-range autocorrelations. Therefore, this study is aimed primarily at assessing LRA, as well as spatiotemporal gait variables, by means of comparison between NW and Usual Walking (UW) in PD. Considering NW may act as an external cue to restore control movement [12], our primary hypothesis is that gait variability would become less random and spatiotemporal gait variables would improve with such a rehabilitative approach. Secondarily, the study is aimed at comparing long-range autocorrelations and gait variables in the PD population to healthy adults while usual and Nordic walking.

## Methods

### Participants

Fourteen PD participants were enrolled from the Neurology Department of the Cliniques universitaires Saint-Luc, Brussels (AJ) and from the “Association Parkinson”, Namur (Table 1). Note that several participants were directly recruited from another previous study which presented the same eligibility criteria [6]. This previous study aimed to investigate the potential usefulness of LRA assessment as a marker of gait instability in PD. A minimum of 6 months has been respected between the two studies. Eligibility criteria included: (1) fulfilment of the United Kingdom Parkinson's Disease Society Brain Bank (UKBB) criteria for idiopathic PD [14] and (2) ability to perform 512 consecutive strides without the need for walking aids. Such series length is required to adequately apply mathematical methods described below [15]. Exclusion criteria comprised of history of other neurologic disorders or orthopaedic pathologies known to impair gait performance. Ten age-matched healthy control participants (Table 1) were recruited as a control group.

**Table 1:** Characteristics of the study populations

	PD (*n* = 14)	Healthy (*n* = 10)	*p*-value
Age (years)	62.2±6.9	60.3±4.8	0.525
Gender (male/female)	9/5	3/7	
Height (cm)	171.8±10.3	170.3±7.6	0.813
Weight (kg)	74.7±15.9	67.8±10.7	0.186
Time since the diagnosis (years)	4.5±2.7	-	
MMSE score (/30)	29.5 [27–30]	30 [29–30]	0.049
H&Y scale	2 [1–3]	-	
1 (*n*=):	2	-	
1,5 (*n*=):	2	-	
2 (*n*=):	5	-	
2,5 (*n*=):	3	-	
3 (*n*=):	2	-	
MDS-UPDRS III (/132)	26.9 [10–46]	-	
MDS-UPDRS total (/260)	52.3 [18–94]	-	
BESTest total (%)	77.4 [69–92]	87.7 [85.2–96]	≤0.001
ABC Scale (%)	79.0 [54–100]	95.0 [72–100]	0.014

Mean (±SD) are expressed for quantitative variables normally distributed while median [range] are expressed for both ordinal and non-normally distributed variables

### Ethics, consent and permissions

Testing took place at the Cliniques universitaires Saint-Luc, Brussels from September 2015 till November 2016. This study was conducted according to the declaration of Helsinki and had ethical approval from the Comité d’Ethique Hospitalo-Facultaire de l’Université catholique de Louvain (B403201318916/Clinical Trial registration: NCT02419768). Participants gave written informed consent prior to data collection.

### Procedure

**Fig. 1:**
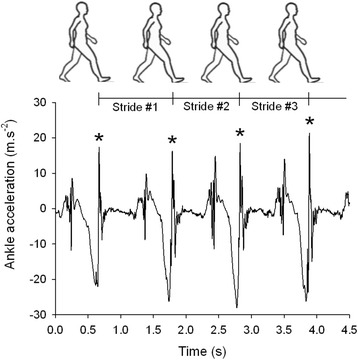
Stride duration determination—Peak detection method. Heel strikes correspond to each positive acceleration peak (*; peak detection method). The time between successive heel contacts corresponds to the stride duration

During UW and NW sessions, a unidimensional accelerometer was taped, in the antero-posterior direction, on to the lateral malleolus of the side most affected by motor symptoms of PD and of the dominant side for healthy participants. Acceleration data was recorded at 512 Hz using the Vitaport three ambulatory recorder (Temec Instruments B.V., Kerkrade, The Netherlands), and transferred to a computer. The stride duration was determined from the peak of acceleration (i.e. peak detection method [16–20]; Fig. 1) detected by the software internally developed and confirmed visually. The visual inspection consisted of checking each acceleration peak detected after the application of the peak detection algorithm to ensure a precise measure of successive stride durations and subsequently its variability. Validated against ground reaction forces, the peak detection method assumes that each acceleration peak corresponds to successive foot contact [16, 18]. The peak detection method was designed to minimize the risk of false step detection making it the most accurate compared with other techniques (e.g. zero-crossing method) [16, 17]. Accelerometer data was collected during a walking session of 12 min that consisted of walking overground at a self-selected speed around a 42 m oval indoor track with and without Nordic poles (usual and Nordic walking sessions). The two walking sessions were performed in a quiet environment to avoid all external perturbations that could increase the attentional cost of walking. Concerning NW, each PD and healthy participant received three 1-h sessions to learn the walking technique (ALFA method). Note that all PD and healthy participants were naive with NW technique prior to the study. The training sessions were delivered 1 week before the experimental sessions. The number of training sessions was limited to three to allow enough time to learn the basic walking technique but not to induce motor learning [10]. Experimental sessions were spread over two consecutive days and at the same part of the day in order to, respectively, reduce the fatigue and medication influences on gait in PD participants [21, 22]. Also, the order of the two walking sessions was randomly allocated in order to avoid any gait rehabilitation benefits. Before the NW session, the walking technique of each patient was verified (MBL). Although the technique learning and familiarization period was respected, no specific instructions on gait pattern were delivered.

Each participant underwent a comprehensive assessment when medication provided significant functional improvement (ON phase), 60–120 min after their last medication intake. The severity of PD (modified H&Y scale), the patient’s functional status (Movement Disorder Society—Unified Parkinson’s Disease Rating Scale (MDS-UPDRS)), and the subjects’ performance on a balance test (BESTest) were assessed and the Activities Balance Confidence questionnaire (Activities-specific Balance Confidence Scale (ABC Scale)) was administered.

### Gait assessment

#### Stride duration variability

Stride duration variability can be investigated either in terms of magnitude, using standard metrics such as sample mean, standard deviation (SD) and coefficient of variation (CV), or in terms of its organization (i.e. LRA), which provides complementary information on how stride duration evolves with time across consecutive strides [23, 24].

##### Temporal organization of the stride duration variability (LRA)

The presence of LRA was evaluated, using an integrated approach that combines the results of Rescaled Range Analysis (Hurst exponent; H) and Power Spectral Density (α exponent). For each time series, both methods were applied to sequences of 512 consecutive gait strides [16]. To increase the level of confidence in the results, the consistency of H and α exponents was verified using the asymptotic relationship *d* = H - [(1 + α)/2] [15]. Following this integrated approach, the following three criteria must be met to conclude the presence of LRA:

- H > 0.5
- α significantly different from 0 and lower than 1
- *d* ≤ 0.10

For further details, these methods are described elsewhere [15].

##### Magnitude of the stride duration variability (CV)

Recently suggested as reflecting the attentional load allocated to the motor task [25], the magnitude of stride duration variability was assessed using the coefficient of variation (CV = [SD/mean] * 100) which was calculated for the 512 consecutive stride previously selected for each time series.

#### Spatiotemporal gait variables

Lap times and the number of laps were measured. The total distance was determined from the number of laps of 42 m performed by the participants. Following the relationship between total walking distance and acquisition duration, mean gait speed, mean gait cadence and mean step length was independently assessed as follow:

- Mean gait speed (m.s^-1^) = Total walking distance (m)/Acquisition duration (s)
- Gait cadence (#steps.min^-1^) = Total number of steps (#)/Acquisition duration (min)
- Step length (m) = Gait speed/Gait cadence

Note that stride duration variability and spatiotemporal gait variables were extracted from 512 consecutive gait strides. This large data number is required to measure gait variability, in particular to draw adequate conclusions about the temporal organization of the stride duration variability [15].

### Transformation of gait data into Z-score

As age and walking speed were shown to influence gait variables [26, 27], individual participant values were normalized to Z-score to identify PD-caused gait alterations as opposed to changes arising solely from the age of subjects or from different walking speeds. Normative data of spatiotemporal gait variables were extracted from the Andriacchi et al. and Winter’s reference works [26, 27]. Given that no normative values for H and α exponents are currently established and that no influence of age and gait speed were demonstrated, values from our healthy control population were used as a reference point. Whereas the influence of gait speed on the CV of stride duration was highlighted at low speeds (i.e. 0.2 to 0.6 m.s^-1^), people with PD with mild and moderate motor symptoms usually walk at a gait speed above 1 m.s^-1^ [6, 23, 25, 28–30]. Thus, considering this aspect and the absence of normative data for the CV of stride duration, healthy control values were also used as a reference point.

### Statistical analysis

Sample size calculation was performed for the Hurst exponent as the main outcome. A sample size of 14 participants (power = 0.8, alpha = 0.05) was required to detect similar differences as the study of Hove et al. that investigated the influence of the rhythmic auditory stimulation on the temporal organization of the stride duration variability in PD [31].

Anthropometric and clinical characteristics were analysed with independent samples *t*-tests. A paired *t*-test and a two-way ANOVA (Walking condition x Pathological condition) repeated measure were applied to examine the influence of walking condition, as well as one of the pathological condition on LRA exponents, CV of stride duration and spatiotemporal gait variables. Holm-Sidak post hoc tests were performed and the effect size (partial eta squared; *η* ^2^_*p*_) was measured. One-sample *t*-test or Wilcoxon signed-rank test (non-normally distributed variables) against zero was used to compare subjects Z-transformed gait variables with normal values (corresponding to Z score = 0). The results were considered statistically different for *p*-values < 0.05.

## Results

Anthropometric and clinical characteristics of the fourteen PD participants and the ten healthy controls are summarized in Table 1. PD participants presented mild to moderate motor symptoms, corresponding to stage one to three on the modified Hoehn & Yahr scale. Two patients suffered from motor fluctuations. However, none of them presented such fluctuations during the two walking sessions. The two groups were matched for age and height. PD participants reached significantly lower scores in balance performance and confidence compared to healthy controls.

### Stride duration variability

**Fig. 2:**
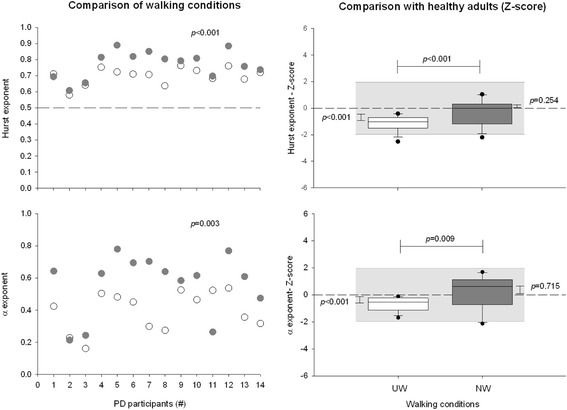
Temporal organization of stride duration variability. Comparison of Hurst and α exponents on usual walking (UW; ○) and Nordic walking (NW; ) in PD and comparison with healthy adults (Z-score)

**Fig. 3:**
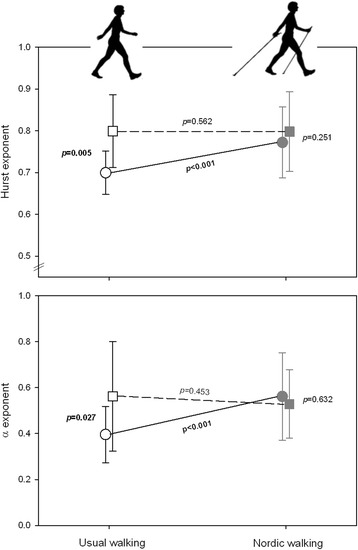
Influence of pathological and walking conditions on Hurst and α exponents in PD and healthy participants. Post-hoc analysis for the (Pathological x Walking) interaction for LRA exponents in PD (*white and grey circles* for usual and Nordic walking sessions, respectively; *black solid line*) and healthy controls (*white and grey squares* for usual and Nordic walking sessions, respectively; *black dashed line*)

**Fig. 4:**
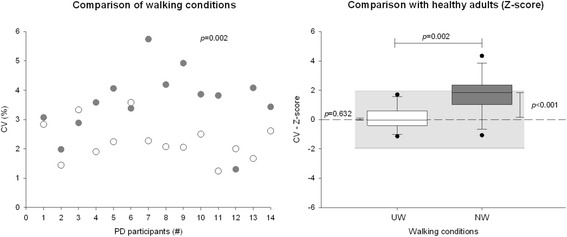
Magnitude of stride duration variability. Comparison of coefficient of variation of stride duration variability (CV) on usual walking (UW; ○) and Nordic walking (NW; ) in PD and comparison with healthy adults (Z-score)

The temporal organization and the magnitude of the stride duration variability are presented in Figs. 2, 3 and 4 and in Table 2.

**Table 2:** Absolute mean values of the stride duration variability and spatiotemporal gait variables for the comparison between the usual walking (UW) and the Nordic walking (NW) sessions in Parkinson’s disease and healthy controls and analysis of the (Pathological x Walking conditions) interaction for the stride duration variability and spatiotemporal gait variables

	Parkinson’s disease		Healthy controls		Interaction	
	UW	NW	UW	NW	*p*-value	*η* ^2^_*p*_
Stride duration variability						
Temporal organization						
H exponent	0.70 (±0.05)	0.77 (±0.08)***	0.80 (±0.09)^§§^	0.81 (±0.08)	**0.012**	0.252
α exponent	0.39 (±0.12)	0.56 (±0.19)**	0.56 (±0.22)^§^	0.52 (±0.14)	**0.002**	0.346
Magnitude						
CV (%)	2.26 (±0.66)	3.59 (±1.11)**	2.17 (±0.82)	2.92 (±0.91)*	0.255	0.058
Spatiotemporal gait variables						
Gait speed (m/s)	1.18 (±0.19)	1.26 (±0.24)	1.47 (±0.17)	1.59 (±0.14)**	0.468	0.025
Gait cadence (#steps/min)	112.90 (±9.48)	102.83 (±13.16)^**^	119.22 (±4.55)	117.15 (±5.47)*	0.063	0.155
Step length (m)	0.63 (±0.09)	0.73 (±0.09)***	0.74 (±0.09)	0.81 (±0.08)**	0.370	0.038

Absolute value is expressed as mean (± SD)

***: *p* ≤ 0.001; **p ≤ 0.01; *p ≤ 0.05 for the comparison between UW and NW (paired *t*-test)

§§§: *p* ≤ 0.001; §§ *p* ≤ 0.01; § *p* ≤ 0.05 for the comparison of walking conditions between PD and healthy controls (independent *t*-test)

Bold data indicates if (pathological x walking condition) interaction was significant (*p* < 0.05)

*η* ^2^_*p*_: partial eta squared

#### Temporal organization of the stride duration variability (LRA)

The integrated approach showed the presence of LRA in both UW and NW in all the subjects (Fig. 2 and Table 2). All values of the H exponent were greater than 0.5, the α exponent was always significantly different to 0, and all values of *d* were far below 0.10 (0.02 ± 0.03 and 0.02 ± 0.03 for UW and NW, respectively).

However, in PD participants, highly statistically significant differences were observed between the two walking conditions (*p* ≤ 0.001 and *p* = 0.003 for H and α exponents, respectively; Fig. 2), H and α exponents being lower in UW compared to NW (i.e. closer to 0.5 and 0 for H and α exponents, respectively). Conversely, the walking conditions had no effect on H and α exponents among healthy controls. Importantly, a significant (Walking condition x Pathological condition) interaction (F = 7.421; *p* = 0.012 for H exponent, and F = 11.643; *p* = 0.002 for α exponent) was demonstrated, as illustrated in Fig. 3 and reported in Table 2. Post-hoc comparisons confirmed that the use of Nordic poles influenced exclusively the PD participants’ gait (Fig. 3). LRA exponents values collected from UW were significantly lower than that of healthy adults (*p* ≤ 0.001 for both exponents; Figs. 2 and 3 and Table 3), while they did not differ during the NW session, which suggest an improvement in the temporal organization of the gait pattern with the use of Nordic poles compared to the more randomness variability observed in UW.

**Table 3:** Mean values of the normalized stride duration variability and spatiotemporal gait variables (Z-score) for the comparison between the usual walking (UW) and the Nordic walking (NW) sessions in Parkinson’s disease

	Z-score			
	UW	*p*-value	NW	*p*-value
Stride duration variability				
Temporal organization				
H exponent	−1.14 (±0.60)	**≤0.001**	−0.31 (±0.97)	0.254
α exponent	−0.70 (±0.51)	**≤0.001**	0.49 [−0.59; 0.56]	0.715
Magnitude				
CV (%)	0.11 (±0.81)	0.632	1.72 (±1.35)	**≤0.001**
Spatiotemporal gait variables				
Gait speed (m/s)	−1.51 (±1.33)	**≤0.001**	−0.97 (±1.67)	**0.049**
Gait cadence (#steps/min)	−0.94 (±1.71)	0.059	−2.99 [−3.51; −0.13]	**≤0.001**
Step length (m)	−0.65 (±0.38)	**≤0.001**	0.09 (±0.43)	0.406

Z-score is expressed as mean (± SD) (one-sample *t*-test) or median [range] (one-sample Wilcoxon signed-rank test)

Bold data indicates if the Z-score significantly differed from 0 (*p* < 0.05): One-sample *t*-test (normally distributed values) or one-sample Wilcoxon signed-rank test (non-normally distributed values)

#### Magnitude of the stride duration variability (CV)

Regarding the stride duration fluctuation magnitude, the values of CV were statistically higher during NW than during UW for both populations (*p* = 0.002 and *p* = 0.045 for PD and healthy participants, respectively) (Fig. 4 and Table 2). Importantly, no significant (Walking condition x Pathological condition) interaction was observed (F = 1.366; *p* = 0.255), confirming that the use of Nordic poles influenced similarly the CV of both PD and healthy participants. In comparison to the healthy population, CV is higher with the use of Nordic poles (*p *≤ 0.001) while CV was similar to the healthy population without Nordic poles (Table 3), which can suggest a more attention-challenging task for PD participants.

### Spatiotemporal gait variables

**Fig. 5:**
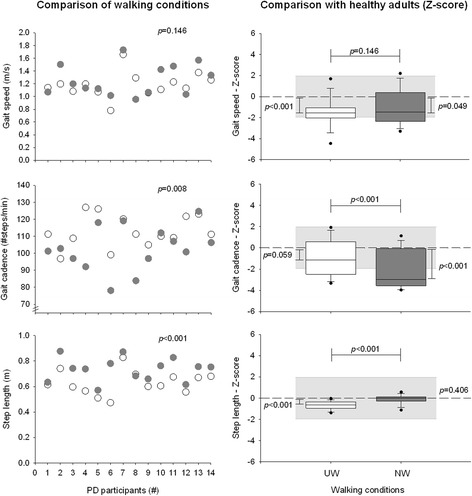
Spatiotemporal gait parameters. Comparison of gait variables on usual walking (UW; ○) and Nordic walking (NW; ) in PD and comparison with healthy adults (Z-score)

Mean absolute and Z-score values for gait speed, cadence and step length are summarized in Fig. 5 and Tables 2 and 3.

While the gait speed remained similar between the two walking conditions (*p* = 0.146), the use of Nordic poles significantly improved the step length (*p* ≤ 0.001) and significantly reduced the gait cadence (*p* ≤ 0.001) in comparison to the UW session (Table 2 and Fig. 5). No significant (Walking condition x Pathological condition) interaction was observed (F = 0.547; *p* = 0.468, F = 3.854; *p* = 0.063, F = 0.839; *p* = 0.370 for gait speed, cadence and step length, respectively), suggesting a similar influence of NW on spatiotemporal gait variables for both PD and healthy participants. Compared to the healthy population, PD participants demonstrated a statistically significant reduction in gait speed (*p* ≤ 0.001) during UW as well as a reduced step length (*p* ≤ 0.001), while gait cadence remained similar to healthy adults. During NW, the gait speed was slightly reduced (*p* = 0.049) as well as the gait cadence (*p* ≤ 0.001) whereas the step length was significantly improved.

## Discussion

In this study, we aimed to gain more insight into the immediate effect of Nordic walking on the temporal organization of gait variability (i.e. LRA) as well as on spatiotemporal gait variables in PD participants. Actively involving rhythmic movements of the upper body, Nordic walking (NW) leads to significant improvement of LRA compared to the more random gait pattern highlighted during the usual walking session (UW). Additionally, NW enhances step length without any change in gait speed. Interestingly, LRA and step length collected from the NW session are similar to that of the healthy population. Those findings strongly support resorting to the use of Nordic poles in the struggle against the randomness of PD gait and the typical gait hypokinesia.

The randomness of PD gait pattern on UW contrasts with the structured gait pattern of age-matched healthy adults. Indeed, stride duration variability on UW appears less structured in PD (i.e. lower LRA exponent values). LRA, highlighted in gait and other rhythmic physiological signals, are thought of as the signature of subtle and complex interactions between subcomponents constituting dynamical systems [9, 32]. Locomotor systems ranks among those systems that need to continuously adapt their pattern to constraints imposed by daily living activities [8]. The randomness of PD gait pattern could thus result from the defective activity among interacting subcomponents (e.g. basal ganglia). Furthermore, the breakdown of LRA highlighted in the specific context of basal ganglia disorders (e.g. Parkinson’s and Huntington’s diseases) was strongly associated with balance and functional status, and was suggested as a quantitative clinical measure of dynamic instability [6, 33].

Compensating or restoring interactions among multiple components involved in healthy gait pattern is a key factor in restoring adaptability and flexibility of locomotion. Interestingly, NW can modulate some temporal organization in PD gait pattern (Fig. 3). LRA exponents were indeed significantly higher with Nordic poles than with usual walking in PD participants and similar to that of the healthy population, characterized by functional and adaptive locomotion (Tables 2 and 3 and Figs. 2 and 3). The use of Nordic poles allows a more temporally organized behaviour than with UW, which suggest a more effective and powerful coordination of sub-components constituting the locomotor system [8, 9, 32].

Note that the α exponent seemed to decrease between the two walking conditions in healthy adults (Table 2). However, the exponent value remained at a “healthy” level (Mean α exponent ± SD: 0.56 ± 0.17) [34]. In addition, such eventual decrease of LRA should be considered within the integrated approach applied in our study. While the effect size is small (Cohen’s d = 0.21) and non-statistically significant (*p* = 0.453) for the α exponent in healthy population, no effect size was demonstrated for the Hurst exponent (Cohen’s d = 0.12; *p* = 0.562). On the other hand, the increase of α exponent is large and highly significant (Cohen’s d = 1.07; *p* < 0.001) in PD participants and evolves in the same way that H exponent (Cohen’s d = 1.05; *p* < 0.001). Therefore, the use of Nordic poles is beneficial for the PD walking pattern whereas it does not affect the walking pattern of healthy population.

Modulation of spatio-temporal gait parameters also seems different for people with PD walking with and without the Nordic poles (Tables 2 and 3 and Fig. 5). Although the gait speed in UW and in NW remained slower than in healthy adults, the overall modulation of spatio-temporal gait parameters with the Nordic poles was similar to healthy adults. While the reduced gait speed during UW was largely attributed to hypokinesia (significant reduction of step length), the gait speed in NW is modulated by both a significant improvement in step length and a reduction in gait cadence, which is particularly interesting in PD gait disorders management. Although the reduction of gait cadence could be perceived as dramatic, it should be noted that such decrease is usually observed with attentional strategies that specifically focus on step length criterion [28, 35].

Decreased gait speed in PD is indeed largely attributed to difficulties in generating appropriate stride length while cadence control, used as a compensatory mechanism, remains intact [1–4]. However, Morris et al. demonstrated that the ability to generate a normal stepping pattern is not lost in PD [2]. Normal movement amplitude could be indeed elicited in PD given the appropriate conditions such as the use of attentional resources or external cues [2, 28]. Both strategies appear to share the same mechanism of focusing attention on the stride length criterion [2].

Attention involvement in gait control on NW could be highlighted by a higher magnitude of gait variability (i.e. CV of stride duration variability; Fig. 4), as recently suggested by Ayoubi et al. in their meta-analysis [25]. Attentional strategies are indeed often used to compensate for the impaired automaticity and could be involved in NW [2, 36]. People with PD need to continuously think about their movement, sequencing the whole movement into sub-movement and the use of the frontal cortex facilitates movement size and timing regulation [36]. During NW, both PD and healthy participants were implicitly focused on walking and their attention was directed to the desired movement patterns [12]. The higher magnitude of the stride duration variability observed in all participants could be the signature of the attention involved in the task being performed, which could be observed with novice participants [37]. In addition, walking with Nordic poles involves the intentional action to move upper limbs in a coordinated fashion with legs, improving the movement of the most affected side in PD.

Therefore, NW appears to be an interesting rehabilitative option as arm swing is reduced in PD [38]. Recent studies agree wholeheartedly with the usefulness of arm swing on the stability of gait, especially in the elderly [38, 39]. Moreover, gait seems to be more stable when the arm swing is amplified and could normalize the gait speed and the step length in this population [38]. As already highlighted by Punt et al., this finding is important as the fall risk is already high for the elderly, and even more so in PD [39, 40]. From this point of view, including arm swing in rehabilitation programmes, it seems to be an interesting strategy to normalize the PD gait pattern [38, 41]. In particular, it has been suggested that normalizing coordination between limbs could improve gait in people with PD [38].

Arm swing is integrated into locomotion via tight neuronal interlimb coordination using specialized neural circuits in the spinal cord (central pattern generators, CPGs) [42]. Although such neuronal networks are able to produce coordinated motor patterns independently, the CPG output can be modulated by afferent feedback (i.e. cutaneous, joint and muscle input) and/or supraspinal input (i.e. cortical control) [41–45]. Interestingly, it has been demonstrated that cutaneous stimulation of the hand evoked significant changes in lower limb kinematics in healthy adults, with an increase in dorsiflexion at the stance-swing transition [46].

In addition, to help focus attention on walking, the use of Nordic poles could also act as an external sensory cue providing the necessary trigger in PD to bypass the defective pallidocortical circuit [12]. Among multiple cueing modalities, auditory and visual cues are the most studied [47, 48]. However, tactile and proprioceptive cues have also demonstrated their usefulness in the struggle against the impaired gait automaticity, despite suggestions of impaired somatosensory function in PD [3].

From a biomechanical point of view, the use of Nordic poles implies a mechanically different movement pattern than UW [49–51]. By increasing the base of support, the use of poles helps to preserve gait stability after an unpredictable perturbation [52]. Nordic poles could serve as walking aids that are commonly prescribed to maintain balance in people with PD. However, the propulsive use of poles could reduce any alteration of the gait pattern classically observed with usual walking aids [49, 53, 54]. Also, higher impulse values and higher knee and ankle moments in the sagittal plane compared to UW could indicate a more dynamic movement in NW [50, 51].

Although the use of Nordic poles seems to be effective in PD, our results should be carefully extended to the whole disease severity spectrum. As recently highlighted, people with PD with only slight and mild gait impairment benefit most from NW [55]. The coordination between limbs during gait seems to be compromised of both bradykinesia and rigidity, which increase with the pathological progression, while the adaptive abilities of arm-leg coordination is preserved in people with PD in the early stage of the disease [56]. Also, people with PD often resort to cognitive strategies to compensate for a lack of gait automacity [57]. However, such compensatory strategies are useful as long as the cognitive resources are preserved [11, 57]. The attentional involvement on gait induced by the use of Nordic poles could, as a result, be thus compromised in people with PD in the latest stages of the disease.

Some limitations of this study should be addressed. Motor complications, such as freezing of gait or dyskinesia, could alter the gait dynamics. However, only two patients suffered from such complications (one from freezing of gait and one from dyskinesia of the upper limb) and none of them presented such complications during the walking trial. Also, note that participants were probably the most motivated since they already participated in a previous study. Thus, our results should be interpreted in light of this specific aspect. To better characterize the gait rhythmicity, ankle accelerometry was preferred to other locations since a significant degree of attenuation of the heel strike transient occurs during the passage to the head [58]. Acceleration peaks are indeed higher and straighter in ankles than the trunk, or even more the head, which could improve the peak detection method by minimizing false-acceleration peak detection. Hence, the results concern the temporal organization of stride duration variability measured from accelerations of the ankles and comparisons with other studies should take into account that other studies extract gait variability from different devices (foot switches [5, 22], inertial sensors [59], accelerometer [34], force platforms [60], optoelectric system [61]) and other body parts (trunk [17], knee [61], ankle [34]) to assess LRA.

While several clinical trials were recently conducted to study the influence of NW on both motor and non-motor symptoms in PD [10–12], none assessed the beneficial effects of NW program on LRA, as a gait stability index. This study can constitute a pilot study offering new perspective to better characterize the sensitivity of the temporal organization of gait variability to rehabilitation approaches. Thus, it would be interesting to investigate the effectiveness of a tailored NW program on gait stability, concurrently assessed with clinical balance measures in a randomized control clinical trial.

## Conclusion

In addition to the improvement of the typical gait hypokinesia, this study demonstrates in PD that the temporal organization of gait variability, which is considered as a marker of healthy locomotion, could be modulated while using the Nordic poles. Involving a voluntary intersegmental coordination, such improvement could also be due to the upper body rhythmic movements acting as a rhythmic external cue to bypass the defective basal ganglia circuitries of PD. Therefore, Nordic walking may constitute a powerful way to manage gait disorders in PD, while being an accessible and affordable physical activity which offers the advantage that the activity can be performed by everybody, everywhere and at almost any time.

## Abbreviations

ABC Scale: Activities-specific Balance Confidence Scale
BESTest: Balance Evaluation System Test
CPG: Central Pattern Generator
CV: Coefficient of variation
H&Y scale: Hoehn & Yahr scale
LRA: Long-range autocorrelations
MDS-UPDRS: Movement Disorder Society-Unified Parkinson’s Disease Rating Scale
MMSE: Mini Mental State Examination
NW: Nordic Walking
PD: Parkinson’s disease
UW: Usual Walking
